# Extracellular vesicle cross-talk between pulmonary artery smooth muscle cells and endothelium during excessive TGF-β signalling: implications for PAH vascular remodelling

**DOI:** 10.1186/s12964-019-0449-9

**Published:** 2019-11-08

**Authors:** Fernando de la Cuesta, Ilaria Passalacqua, Julie Rodor, Raghu Bhushan, Laura Denby, Andrew H. Baker

**Affiliations:** 1 Centre for Cardiovascular Science, Queen’s Medical Research Institute, University of Edinburgh, 47 Little France Crescent, EH16 4TJ, Edinburgh, EH16 4TJ UK; 2 Present affiliation: Yenepoya Research Centre, Yenepoya University, Deralakatte, Mangalore, India

**Keywords:** Cell communication, Comparative transcriptomics, Cre-loxP, Extracellular vesicles, In vitro imaging, Pulmonary artery, RNA-Seq, TGF-β

## Abstract

**Background:**

Excessive TGF-β signalling has been shown to underlie pulmonary hypertension (PAH). Human pulmonary artery smooth muscle cells (HPASMCs) can release extracellular vesicles (EVs) but their contents and significance have not yet been studied. Here, we aimed to analyse the contents and biological relevance of HPASMC-EVs and their transport to human pulmonary arterial endothelial cells (HPAECs), as well as the potential alteration of these under pathological conditions.

**Methods:**

We used low-input RNA-Seq to analyse the RNA cargoes sorted into released HPASMC-EVs under basal conditions. We additionally analysed the effects of excessive TGF-β signalling, using TGF-β1 and BMP4, in the transcriptome of HPASMCs and their EVs. We then, for the first time, optimised Cre-loxP technology for its use with primary cells in vitro, directly visualising HPASMC-to-HPAEC communication and protein markers on cells taking up EVs. Furthermore we could analyse alteration of this transport with excessive TGF-β signalling, as well as by other cytokines involved in PAH: IL-1β, TNF-α and VEGFA.

**Results:**

We were able to detect transcripts from 2417 genes in HPASMC-EVs. Surprisingly, among the 759 enriched in HPASMC-EVs compared to their donor cells, we found Zeb1 and 2 TGF-β superfamily ligands, GDF11 and TGF-β3. Moreover, we identified 90 genes differentially expressed in EVs from cells treated with TGF-β1 compared to EVs in basal conditions, including a subset involved in actin and ECM remodelling, among which were bHLHE40 and palladin. Finally, using Cre-loxP technology we showed cell-to-cell transfer and translation of HPASMC-EV *Cre* mRNA from HPASMC to HPAECs, effectively evidencing communication via EVs. Furthermore, we found increased number of smooth-muscle actin positive cells on HPAECs that took up HPASMC-EVs. The uptake and translation of mRNA was also higher in activated HPAECs, when stimulated with TGF-β1 or IL-1β.

**Conclusions:**

HPASMC-EVs are enriched in RNA transcripts that encode genes that could contribute to vascular remodelling and EndoMT during development and PAH, and TGF-β1 up-regulates some that could enhance this effects. These EVs are functionally transported, increasingly taken up by activated HPAECs and contribute to EndoMT, suggesting a potential effect of HPASMC-EVs in TGF-β signalling and other related processes during PAH development.

## Background

Pulmonary arterial hypertension (PAH) is a rare disease, with an estimated prevalence which ranges from 10 to 52 cases per million [1]. The development of PAH is associated with aggravation of clinical symptoms and increased mortality [2]. Alteration of TGF-β signalling is a major cause underlying PAH, as mutations in receptors in the TGF-β superfamily are responsible for almost all familial PAH, with BMPR2 alterations being the most prominent [3]. Although TGF-β and BMP ligands stimulate opposing signalling pathways [3], the regulation of this signalling is complex frequently displaying cross-stimulation [4, 5]. The imbalance of this signalling is the main trigger for the altered phenotype of pulmonary artery cells during PAH [5, 6].

**Fig. 1:**
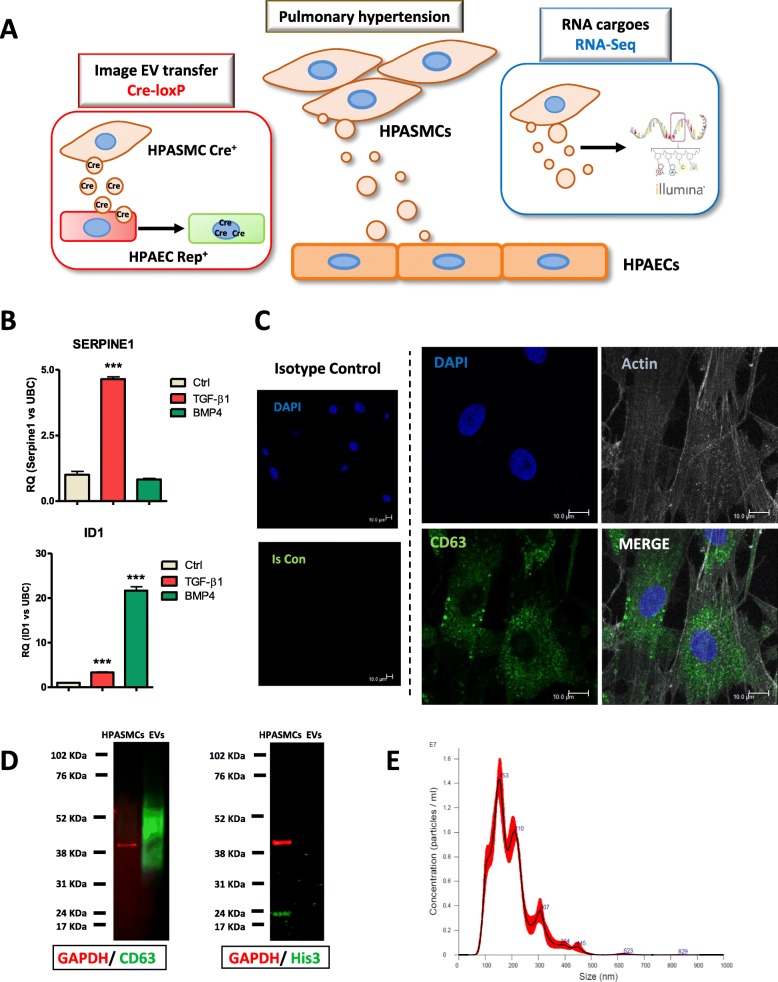
Characterisation of EVs released by HPASMC in vitro. **a**, Schematic representation of the approach used to analyse EVs-mediated HPASMC-to-HPAECs communication. **b**, Quantification of the downstream gene Serpine1 by qRT-PCR shows specific activation with TGF-β1. Id1 was highly activated by BMP4 and mildly by TGF-β1 due to these signalling pathways´ crosstalk, as expected. **c**, Immunocytochemistry showing CD63+ EVs from HPASMCs in cell culture (representative image from *n* = 4 independent experiments). **d**, Identity of HPASMC-EVs isolated by ultracentrifugation was confirmed by expression of CD63 marker, as opposed to their donor HPASMCs. Histone 3 used as a control for cell contamination was only detectable in HPASMCs. GAPDH was used as HPASMC loading control. **e**, The HPASMC-EVs isolated were analysed by Nano Tracking Analysis (NTA) which showed EVs ranging 80–500 nm with a highest peak at 150 nm

Extracellular vesicles (EVs) are essential mediators of cell-to-cell communication [7, 8]. These vesicles contain RNA, lipids, proteins and fragments of DNA [7]. EVs can act as positive or negative modulators of cardiovascular diseases depending on the type and state of the cells from which they can originate. For example, EVs contribute to atherosclerosis progression and plaque rupture promoting microcalcification [9, 10] and, in contrast, certain EVs have beneficial effects on vascular function and endothelial regeneration [11, 12]. In the context of pulmonary function and disease, cross-talk occurs between the two major cell types: endothelial and smooth muscle cells [5]. While transport from human pulmonary arterial endothelial cells (HPAEC) to human pulmonary arterial smooth muscle cells (HPASMCs) has been studied in PAH [13–17], the relevance of HPASMC-to-HPAEC communication remains unclear. Recently, we showed EV-mediated transfer of miR-143 from HPASMC to HPAECs leads to migration and angiogenesis [18]. We therefore hypothesised that imaging HPASMC-EVs-mediated transfer and quantification of their cargo might unveil novel insights through which HPASMCs and HPAECs could impact PAH (Fig. 1 a).

A novel methodology based on Cre-loxP has been recently shown to monitor EV-mediated transfer of mRNA from donor to recipient cells [19, 20]. This methodology was developed to visualize EVs derived from cancer cells and successfully used with progenitor cells from mouse retina [21]. Here, we attempted to adapt the current technology to allow efficient *Cre*-induced recombination in primary vascular cell:cell communication. Here, we show that HPASMC-EVs are enriched in Zeb1 and the TGF-β superfamily ligands GDF11 and TGF-β3. Besides, we found an upregulation of palladin and bHLHE40 with TGF-β1-mediated pathogenic stimulation and provide essential new evidence of a functional EV-mediated transport of mRNA from HPASMC-to-HPAEC and showing increase uptake and translation in activated HPAECs.

## Methods

*An extended version of the Methods section can be found in Supplementary Material.*

### Cell culture

Cultures of HPASMCs and HPAECs have been described previously [18]. Stimulation of HPASMCs with TGF-β1 (10 ng/ml), BMP4 (10 ng/ml) and IL-1β (10 ng/ml) for 48 h renewing stimuli every 24 h. FBS used was depleted of EVs by overnight ultracentrifugation at 100,000·g.

### Isolation of EVs

Supernatants were centrifuged at 2000·g for 10′ at 4 °C to remove dead cells. Then EVs were isolated by means of two steps of ultracentrifugation at 100,000·g for 1 h at 4 °C in a Sorvall WX+ Ultracentrifuge using a TH-641 Swinging Bucket rotor (Thermo Fischer Scientific).

### Western blot

SDS-PAGE and transfer onto nitrocellulose membranes were performed in a Bolt Mini Gel Tank electrophoresis unit (Thermo Fisher Scientific). Primary antibodies used were mouse monoclonal to CD63, ab59479; rabbit polyclonal to Histone 3, ab70550, from Abcam; goat polyclonal to Zeb1, sc-10,572, from Santa Cruz Biotechnology; and mouse monoclonal to GAPDH, D4C6R, and rabbit monoclonal to GAPDH, 14C10 from Cell Signaling Technology. Secondary antibodies used were from LI-COR: IRDye 800CW goat anti-mouse, IRDye 680RD goat anti-rabbit, IRDye 680RD goat anti-mouse and IRDye 800CW goat anti-rabbit. Detection was performed in an Odissey imaging system (LI-COR).

### Nanoparticle tracking analysis (NTA)

The pellet from EVs isolated by ultracentrifugation was suspended in PBS and visualised in a NanoSight LM14 instrument (Malvern Panalytical). An amount of 3–5 videos of 60 s were acquired per sample and only included if exceeding 500 particle tracks. EVs/ml were calculated as average of these technical replicates and normalised to the number of cells counted at endpoint to calculate EVs/cell.

### RNA-Seq

HPASMCs and EVs from supernatants were collected and RNA was extracted with miRNeasy kit (Qiagen), quantified using Qubit technology (Thermo Fisher Scientific) and samples’ quality was checked in a 2100 Bioanalyzer (Agilent, Additional file 1: Fig. S1B). The total amount of RNA obtained from EVs was 9 ng. Thus, library was prepared with SMARTer Low-Input Strand-Specific Total RNA-Seq for Illumina (Takara, Clontech). NGS was performed by BGI Tech Solutions on a HiSeq 4000 (Illumina). The sequencing quality was assessed using FastQC. Number of paired-end reads were between 5.5 × 10^7^–6 × 10^7^ for all samples (Additional file 1: Fig. S1 C). Read mapping to the human genome was carried out with STAR [22] using GENCODE GRCh38 genome sequence and GENCODE v26 transcriptome annotation. To study differential expression at the cell level, we quantified gene expression using HTSeq-count [23], based on the mapped reads. As expected with low-input library sequencing [24], we noted a high level of duplication, in particular for EV samples. To study the transcriptome of EVs, we decided to add a step to remove duplicated reads before the HTSeq-count quantification analysis. The removal of duplicates was done using the markdup option from SAMtools [25]. RNA-Seq data has been submitted to NCBI's Gene Expression Omnibus and are accessible through GEO Series accession number GSE131998. The gene ontology analyses was done using topGO on differential expressed genes over a background of expressed genes [26]. Interaction analyses were performed with String Database v10.5. The interactions calculated by the software include direct (physical) and indirect (functional) associations and are based on genomic context predictions, high-throughput lab experiments, automated text mining and database searching. The network links are not ultimately representative of functional relationships between nodes but rather show a probability of interaction based on the aforementioned calculations.

### Gene expression quantitative real time-PCR (qRT-PCR)

For gene expression analysis, cDNA for mRNA analysis was obtained from total RNA using the RevertAid First Strand cDNA Synthesis Kit (Thermo Fisher Scientific). qRT-PCR was performed using TaqMan primers or Power SYBR green (Life Technologies) with custom PCR primers (Eurofins MWG, primer sequences can be found in Additional file 7: Table S6). Ubiquitin C (UBC) or GAPDH were selected as housekeeping genes due to its stability across all groups studied, as found on the RNA-Seq differential analyses.

### Immunocytochemistry (ICC)

Cells were fixed, permeabilized and incubated with the following primary antibodies: mouse monoclonal to CD9, sc-59,140; mouse monoclonal to CD81, sc-166,029; mouse monoclonal to CD63, ab59479, rabbit polyclonal to BMP11 (=GDF11), ab220951, rabbit polyclonal to TGF-β3, ab15537, from Abcam; and rabbit polyclonal to Zeb1, HPA027524, from Sigma-Aldrich. Phalloidin-iFluor 488 (Abcam) was used to counterstain the cell’s actin cytoskeleton. The secondary antibodies used were: goat anti-rabbit Alexa Fluor 594 (Abcam) and goat anti-mouse Alexa Fluor 647 (Invitrogen). Nuclei were stained with DAPI. Cells were visualized in a TCS SP8 confocal microscope (Leica).

### PKH67 labelling of EVs

EVs were labelled with PKH67 dye for 30 s and then quenched with 1% FBS before amply diluting in FBS-free MEM followed by ultracentrifugation at 100,000×g. The pellet was suspended in FBS-free MEM and EVs/ml were calculated by NTA. 5 × 10^3^ HPASMC-EVs/cell were used to treat HPAECs in culture for 20 h. Ultracentrifugation and addition to HPAECs of a negative control of PKH67 dye alone was carried out in parallel as to assure that aggregated dye or stained protein were not the cause for the observed uptake.

### Production of lentiviral vectors

Lentiviral vectors were produced by triple transient transfection of HEK293T cells as previously described [27]. Lentiviral titres were ascertained by TaqMan quantitative real-time PCR (qRT-PCR) using the primer/probe sequences described on Additional file 7: Table S6. A Cre recombinase lentivirus (LV-Cre) was produced with a LV-CMV-nlsCre plasmid (Plasmid #12265, Addgene: https://www.addgene.org/). A reporter lentivirus (LV-Rep) was generated from the plasmid pLV.CMV.MCS.LoxP-DsRed-Loxp-eGFP, which was a kind gift from Prof. Jacco Van Rheenen, Hubrecht Institute. An empty lentiviral vector was also produced to use as Null control for LV-Cre transduction.

### Primary cell Cre-loxP method

Donor HPASMCs were transduced with LV- Cre and recipient HPAECs with LV-Rep. HPASMCs Cre + and HPAECs Rep+ were co-cultured for 7 days using a Cre+: Rep+ ratio of 3 as to enhance EV transfer. Cre-mediated recombination was calculated by the %eGFP^+^/DsRed^+^ ratio, as measured by FACS. The markers smooth-muscle actin (SMA) and collagen III were quantified at endpoint (7 days) using the antibodies mouse monoclonal SMA-405 (IC1420V, R&D); and rabbit monoclonal collagen III (ab184993, Abcam) with a secondary goat anti-rabbit Alexa Fluor 647 (Invitrogen), after fixing with 2% paraformaldehyde and permeabilising with 0.1% Triton X-100. Stimulation of co-cultures with TGF-β1 (5 ng/ml), BMP4 (5 ng/ml), IL-1β (5 ng/ml), TNF-α (1 ng/ml) and VEGFA (20 ng/ml) during the 7 days protocol was renewed every 48 h.

### Statistical analysis

RNA-Seq differential expression analysis was performed using DESeq2 on the raw read count. Sample clustering was evaluated using the Principal component analysis (PCA) tool available in DESeq2 [28] on the regularized log transformed data. The correlation between the Log fold chance in EV and the Log fold change in Cells after TGF-β1 treatment was assessed using the Pearson correlation test. For the gene ontology analyses, Fisher’s exact test was used to calculate the *p*-values.

Data in graphs are given as mean ± standard error mean (SEM). Comparisons between 2 groups were analysed using 2-tailed unpaired Student’s t test. All statistical analyses of qRT-PCR data were performed on the dCt scale whereas for graphical representation RQ ± SEM was used. For validation analyses of RNA-Seq data performed by qRT-PCR a 1-tailed unpaired Student’s t test was used. No evidence of unequal variances across groups was found for any of analyses of the dCt scale data using Levene’s test prior to statistical analysis. Statistical significance is denoted by a *P* value of less than 0.05 with 3 different categories represented: *P ≤ 0.05, **P ≤ 0.01, ***P ≤ 0.001.

## Results

### Characterisation of HPASMC-derived EVs

We have investigated disease-associated signalling pathways aiming to characterise its potential impact in PAH using cultures of HPASMCs stimulated with or without TGF-β1 or BMP4 (Additional file 1: Fig. S1A). To confirm the activation of associated pathways, we quantified the downstream genes Serpine1 and Id1 (Fig. 1b). We then investigated whether HPASMCs contain EVs under physiological conditions using the EV markers CD9, CD81 and CD63. Staining for CD9 or CD81 was mainly present on plasma membrane (Additional file 1: Fig. S2A), while CD63 EV-like structures showing positive staining to CD63 were found within the cytoplasm and membrane of HPASMCs (Fig. 1c). This is consistent with the main subcellular location found in Human Protein Atlas (vesicle for CD63, membrane for both CD9 and CD81, Additional file 1: Fig. S2B) and for this reason we used CD63 marker for further immunocytochemical (ICC) experiments. HPASMC-derived EVs were efficiently isolated by ultracentrifugation, as verified by protein expression of CD63 and His3 as negative marker (Fig. 1d). NTA further confirmed the presence of EVs, which ranged between 80 and 500 nm (Fig. 1e). Therefore, we efficiently isolated HPASMC-EVs for further molecular analysis.

### HPASMC- derived EVs show a specific transcriptome and are enriched in TGF- β superfamily ligands GDF11, TGF-β3 and transcription factor Zeb1

**Fig. 2:**
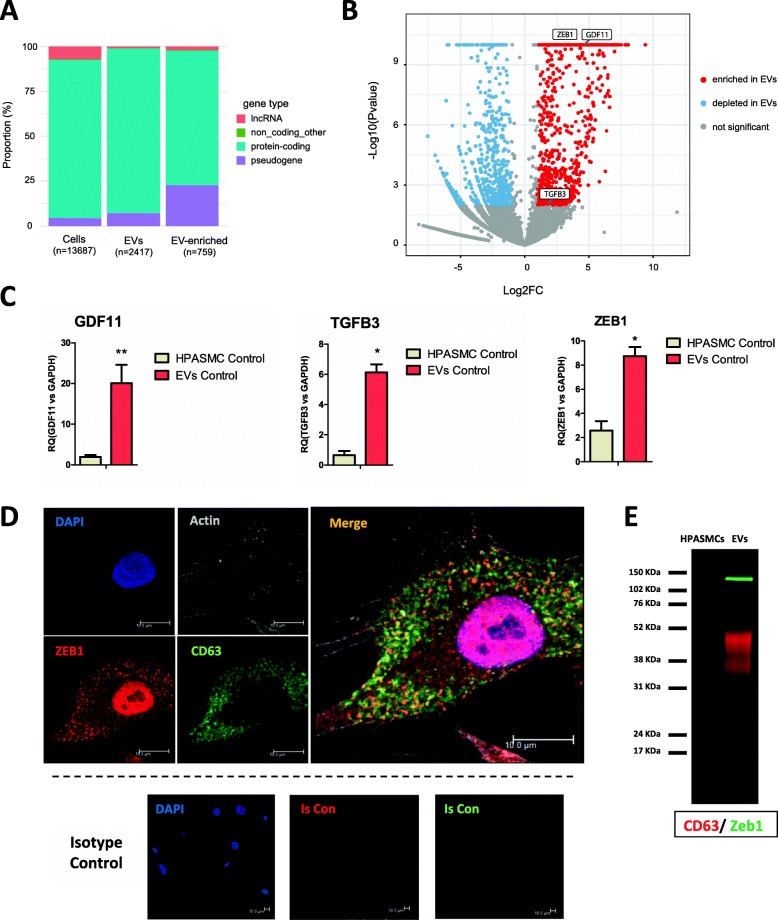
HPASMC-EVs show a specific transcriptome and are enriched in Zeb1, GDF11 and TGF-β3. Data from the low-input RNA-Seq was analysed to examine the cargoes sorted into HPASMC-EVs. Enrichment analysis was performed consisting in the quantification of cargoes with higher expression in EVs compared to their donor HPASMCs (pvalue < 0.05 and FC > 2). **a**, Proportion of coding/non-coding RNAs detected in HPASMC and their EVs. **b**, Volcano plot showing the transcripts depleted or enriched in EVs compared to donor HPASMCs (pvalue < 0.05 and FC < − 2 or FC > 2). Among the enriched we found: Zeb1, GDF11 and TGF-β3. **c**, Validation by qRT-PCR of the enrichment of GDF11, TGF-β3 and Zeb1 in HPASMC-EVs (*n* = 3 biological replicates, data are presented as mean ± SEM. *P ≤ 0.05, **P ≤ 0.01, ***P ≤ 0.001 (Student’s t-test). **d**, Immunocytochemistry of Zeb1 and CD63 proteins showing co-localisation within the cytoplasm of HPASMCs. **e**, Western Blot showing a specific expression of Zeb1 protein in EVs. Scale bars = 10 μm. Protein interaction analyses were performed with STRING and GO enrichment using DAVID software. PCA = principal component analysis, GO = Gene Ontology

In order to analyse the RNA diversity in HPASMCs and their released EVs we used low-input RNA-Seq. We detected transcripts from 2417 genes from HPASMC-EVs and 13,867 in cellular RNA (Fig. 2a). We sought to examine differences in transcriptome of HPASMCs and EVs to determine RNAs specifically sorted into EVs. For that purpose, an enrichment analysis was performed consisting of the quantification of cargoes with higher expression in EVs compared to HPASMCs. 759 RNAs were found significantly enriched in EVs (Additional file 2: Table S1). This implies that EVs contain a specific transcriptome. The majority of RNAs enriched in EVs are protein-coding but there is an overrepresentation of pseudogenes (Fig. 2a). Among the cargoes enriched in healthy HPASMC-EVs, we found GDF11, TGF-β3 and Zeb1 (Fig. 2b). This was validated by qRT-PCR in 3 independent biological replicates (Fig. 2c). We then examined protein levels by ICC on HPASMCs studying co-localization with CD63. In the case of GDF11 and TGF-β3 co-localization with CD63 was negative, with GDF11 being very minimally expressed on the protein level (Additional file 1: Fig. S3A and S2B). Remarkably, Zeb1 protein was found co-localized with CD63 within cytoplasm (Fig. 2d). Moreover, western blot analysis showed a specific expression of Zeb1 protein in EVs, being almost undetectable in HPASMCs, corroborating that the enrichment is occurring at both mRNA and protein levels. The very low levels of Zeb1 in HPASMCs observed by western blot are surprising given the results observed by ICC (Fig. 2d). The reasons for this might be that the extraction of nuclear proteins is not as efficient as the one from cytoplasmic ones and that vesicles arising from membrane might be lost during washes while preparing cells for extraction.

The results obtained show a specific transcriptome of HPASMC-EVs, which are enriched in TGF-β superfamily ligands and Zeb1 in basal conditions.

### Excessive TGF-β signalling promotes switch of HPASMCs towards a pro-fibrotic phenotype and alters EVs’ cargo

**Fig. 3:**
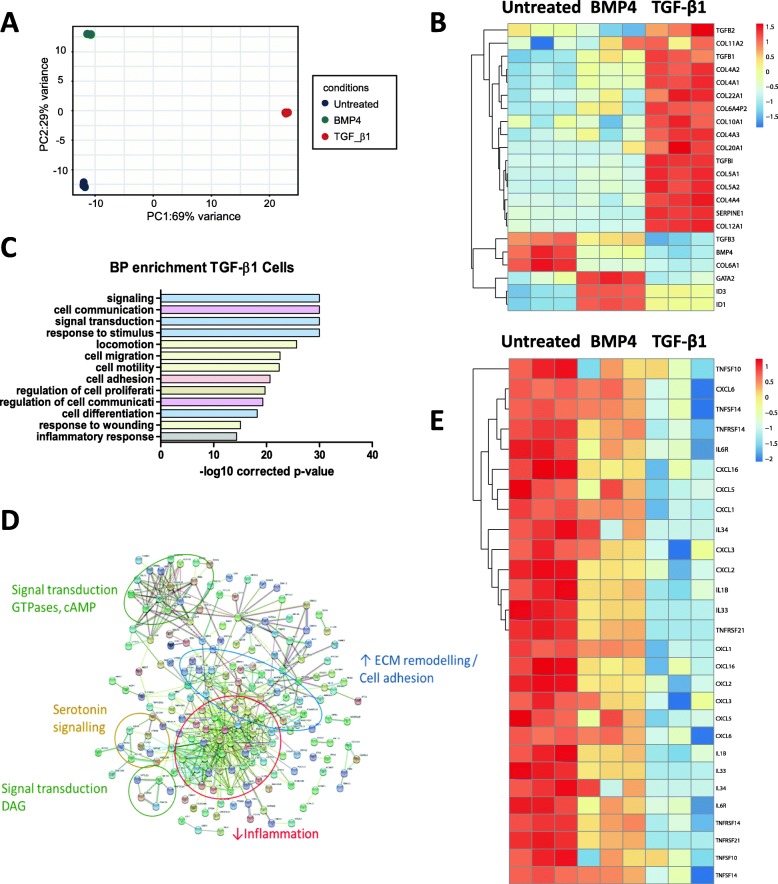
Stimulation of TGF-β signalling triggers fibrotic phenotype and downregulates inflammatory pathways on HPASMCs. **a**, Control, TGF-β1-treated and BMP4-treated HPASMCs were analysed by RNA-Seq. PCA analysis of HPASMCs showed a greater effect of TGF-β1 than BMP4, as these HPASMCs were separated from the rest by the first principal component, accounting for 69% of the variance. **b**, Heatmap showing the activation of specific downstream markers of TGF-β1 and BMP4 pathways. **c**, GO analysis for biological process of the differential transcripts in TGF-β1-treated HPASMCs provided significant terms relevant to PAH: cell migration (yellow), proliferation (brown) and inflammation (grey), as well as with cell communication (pink). **d**, Interactome analysis of the top differential RNAs in TGF-β1-treated HPASMCs. Most relevant clusters are highlighted: Inflammation, ECM remodelling/cell adhesion, serotonin signalling and signal transduction. **e**, Heatmap showing the inflammatory markers downregulated in response to activation of TGF-β signalling in HPASMCs. Treatment of HPASMC with TGF-β1 or BMP4 (both 10 ng/ml) was performed for 48 h and renewed every 24 h. Protein interaction analyses were performed with STRING and GO enrichment using DAVID software. PCA = principal component analysis, GO = Gene Ontology

We next sought to quantify the effect of pathogenic stimuli on HPASMC transcriptome and EVs’ cargo. HPASMCs were treated with or without TGF-β1 or BMP4. At the cellular level, PCA showed a clear separation of the 3 groups and the major variance associated with TGF-β1 treatment (Fig. 3a). Within HPASMCs TGF-β1 treatment resulted in 1484 differential transcripts and BMP4 in 608 (Additional file 3: Table S2). Classical downstream genes were activated with each treatment (Fig. 3b). TGF-β1 induced Serpine 1 and 2, TGF-β1 and TGF-β2, and several collagen variants. BMP4 activated Id1 and Id3, GATA2 [29], and showed a much lower up-regulation of collagens. GO enrichment analysis of TGF-β1 differential genes highlighted some biological processes relevant to PAH such as cell migration, proliferation and inflammation (Fig. 3c and Additional file 4: Table S3). Interestingly, cell communication was significantly represented and could suggest an activation of crosstalk pathways i.e. EV-mediated communication, leading to study EVs more deeply. Pathway analysis reinforced augmented fibrosis signalling with TGF-β1 and showed downregulation of some inflammatory pathways (Fig. 3d) including interleukins, TNF-α receptors and CXCLs (Fig. 3e). The latter was also observed in BMP4 treated HPASMC although to a lower extent (Fig. 3e).

**Fig. 4:**
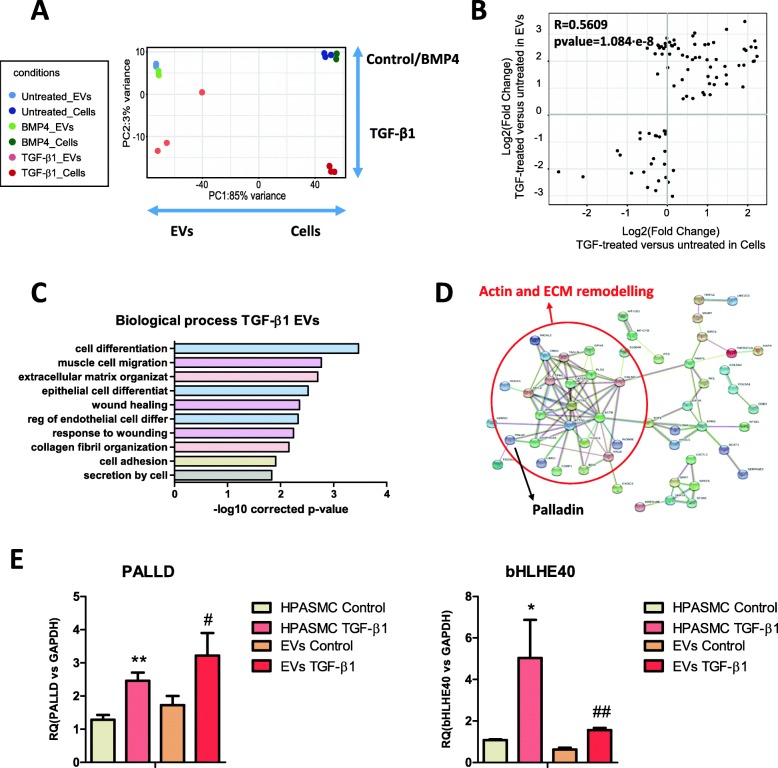
EVs from TGF-β1 treated HPASMCs reflect changes in gene expression and carry greater amounts of palladin and bHLHE40. **a**, Data from the low-input RNA-Seq was analysed to find differentially expressed RNAs present in EVs from HAPSMCs treated with TGF-β1 and BMP4. PCA showed EVs from TGF-β1 treated cells being further separated from controls than those from BMP4-EVs, in a similar extent to their donor cells. **b**, Representation of fold change in TGF-β1 treated HAPSMCs and their EVs showing correlation of the alterations found on differential transcripts. **c**, GO analysis for biological processes performed with the differential transcripts found in EVs from TGF-β1 treated HAPSMCs. **d**, Protein interaction analysis of the RNAs differentially expressed in EVs from TGF-β1-treated HPASMCs compared to control EVs. A node involved in actin and ECM remodelling has been underlined. Position of palladin in this node is highlighted. **e**, Validation by qRT-PCR of the upregulation of palladin and bHLH40 in HPASMCs and their EVs after 48 h TGF-β1 stimulation (*n* = 3 biological replicates). Data are presented as mean ± SEM. *P ≤ 0.05, **P ≤ 0.01, ***P ≤ 0.001 (Student’s t-test). Treatment of HPASMC with TGF-β1 (10 ng/ml) was performed for 48 h and renewed every 24 h. Protein interaction analyses were performed with STRING and GO enrichment using DAVID software. PCA = principal component analysis, GO = Gene Ontology

We next compared the transcriptome of EVs in the three different conditions. EVs from TGF-β1 treated cells showed greater differences compared to control than BMP4, as shown by PCA (Fig. 4a), mirroring what we observed in the cells (Fig. 3a). We found 90 differential RNAs in TGF-β1 EVs, whereas only 5 in BMP4 (Additional file 5: Table S4). For this reason we then focused on TGF-β1 EVs for subsequent analysis. To explore whether levels of expression in EVs reflect gene expression in TGF-β1 treated cells, we performed a correlation analysis of read numbers. The analysis showed a significant correlation (R = 0.5628, *p* value = 1.084·10^− 8^; Fig. 4b). GO enrichment analysis highlighted that the differential RNAs found in TGF-β1 EVs were related to cell differentiation, migration and response to wounding (Fig. 4c and Additional file 6: Table S5) and interaction analysis showed a cluster of RNAs related to actin and ECM remodelling (Fig. 4d). Among these RNAs, we validated palladin and bHLHE40 by qRT-PCR in 3 independent biological replicates (Fig. 4e). Thus, HPASMCs change their phenotype in response to excessive TGF-β signalling and this results in a reflection in the levels of some RNAs in EVs, including upregulation of palladin and bHLHE40.

### EV-mediated transfer of mRNA from HPASMCs is translated into protein in HPAECs and induces overexpression of smooth-muscle-actin

**Fig. 5:**
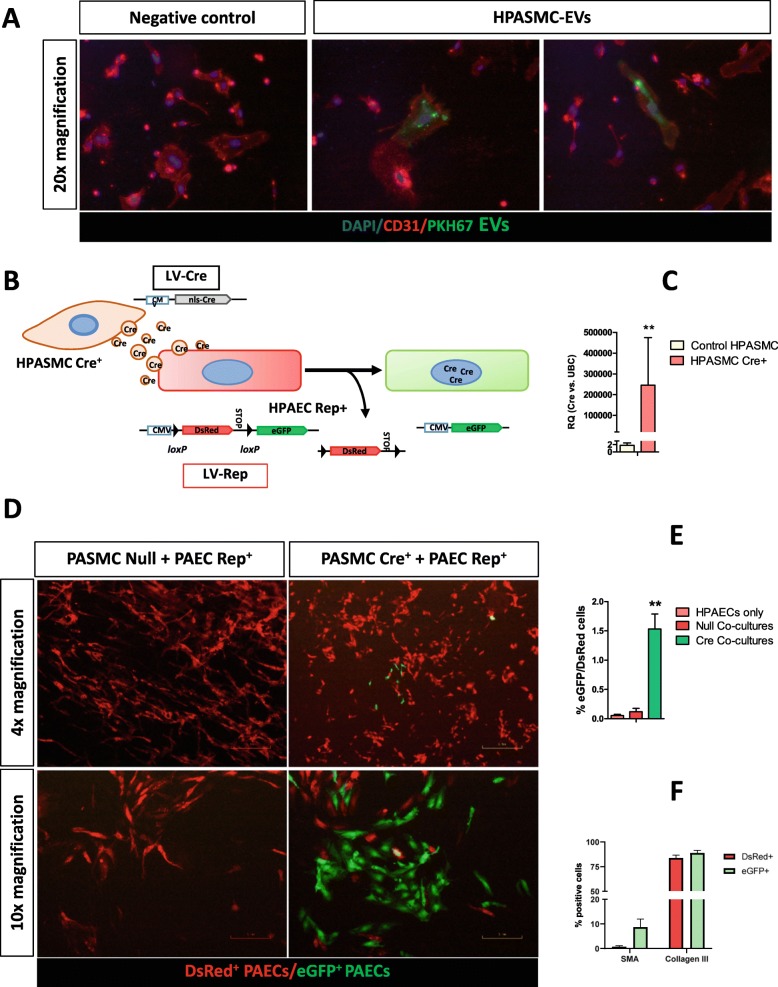
EV-mediated transfer of mRNA from HPASMC to HPAECs results in protein translation in vitro. **a**, HPAECs in culture were treated 20 h with 5 × 10^3^ HPASMC-EVs/cell labelled with PKH67. Then, immunocytochemistry for CD31 was carried out. Many CD31^+^ HPAECs were shown to bind and take up HPASMC-EVs. **b**, Optimised Cre-loxP method for monitoring EV transfer for the first time applicable to in vitro primary cells with limited lifespan. A lentiviral vector was used to transduce donor cells (HPASMC) assuring high efficiency of transgene expression. Recipient cells (HPAECs) were transduced with the original lentiviral reporter construct at a low MOI = 0.1 to avoid background recombination and sorted in a FACS Aria Fusion sorter (BD Biosciences) to provide 100% Reporter+ cells. **c**, Amount of Cre mRNA quantified by qRT-PCR on EVs from HPASMC Cre^+^ and compared to control HPASMCs. Average fold-change above 2 × 10^5^ showed clear induction of the sorting of Cre mRNA into HPASMC-EVs. **d**, Co-cultures of HPASMC Cre^+^ and HPAECs Rep^+^ showed specific recombination on HPAECs switching into green fluorescence, as opposed to negative control HPASMC Null+: HPAECs Rep+ cells co-cultures. **e**, Quantification of Cre-induced recombination by FACS showed significant increase in co-cultures as opposed to control (HPAEC Rep^+^ only) and HPASMC Null^+^: HPAECs Rep^+^ cells co-cultures. **f**, The markers SMA and collagen III were quantified by flow cytometry at day 7 after HPASMC Cre^+^: HPAECs Rep^+^ co-culture. Differential analysis of HPAECs that take up HPASMC-EVs (eGFP^+^) vs. HPAECs that do not (DsRed^+^ eGFP^−^) showed a significant increase of SMA (13-fold, pvalue = 0.016) in eGFP^+^ cells and a mild close-to-significant increase of collagen III (1.06-fold, pvalue = 0.087). Data are presented as mean ± SEM. *P ≤ 0.05, **P ≤ 0.01, ***P ≤ 0.001 (Student’s t-test). All procedures were carried out with n = 3 biological replicates. Scale bars 4x = 300 μm; 10x = 120 μm; 20x = 60 μm. SMA: smooth-muscle actin.

Since EVs from HPASMC in biological and pathological conditions contain RNA with the potential to affect endothelial cell biology, we aimed to confirm their up-take by HPAECs. First, HPASMC-EVs were labelled with PKH67 dye and added to HPAECs in culture. After 20 h, many HPAECs presented green fluorescent particles in their cell membrane (Fig. 5a), confirming the transfer of EVs.

To prove functional transfer of the RNAs contained in EVs, we optimised the recently described Cre-loxP method [20] to make it for the first time applicable to vascular primary cell model systems. For that purpose, the vector chosen to generate Cre^+^ donor cells was a lentivirus (LV-nlsCre) due to its efficient gene delivery to primary vascular cells (Fig. 5b). Following LV-mediated infection of HPASMC, qRT-PCR showed a clear presence of Cre mRNA in HPASMC Cre^+^-EVs (Fig. 5c), showing efficient loading into EVs. HPASMC Cre^+^ and HPAECs Rep^+^ were co-cultured. We observed significant *Cre*-mediated recombination compared to control cultures from HPASMC Null: HPAECs Rep^+^ and HPAECs Rep^+^ cells alone (Fig. 5d). This proves transfer of Cre mRNA from HPASMC to HPAEC and translation into protein in the recipient HPAECs. Two markers related to EndoMT and vascular remodelling were analysed on HPAECs Rep^+^ after 7 days co-culture with HPASMCs Cre^+^, SMA and collagen III. We observed increase of SMA^+^ cells (13-fold, pvalue = 0.016) on HPAECs that took up HPASMC-EVs after co-culture (eGFP^+^), compared to those that did not (DSRed^+^ eGFP^−^), showing induction of the expression of this EndoMT marker by HPASMC-EVs. Collagen III showed a moderate close-to-significance (1.06-fold, pvalue = 0.086) increase on positive HPAECs.

### EV-mediated transfer of mRNA from HPASMC to HPAEC in vitro is enhanced by TGF-β1 and IL-1β

**Fig. 6:**
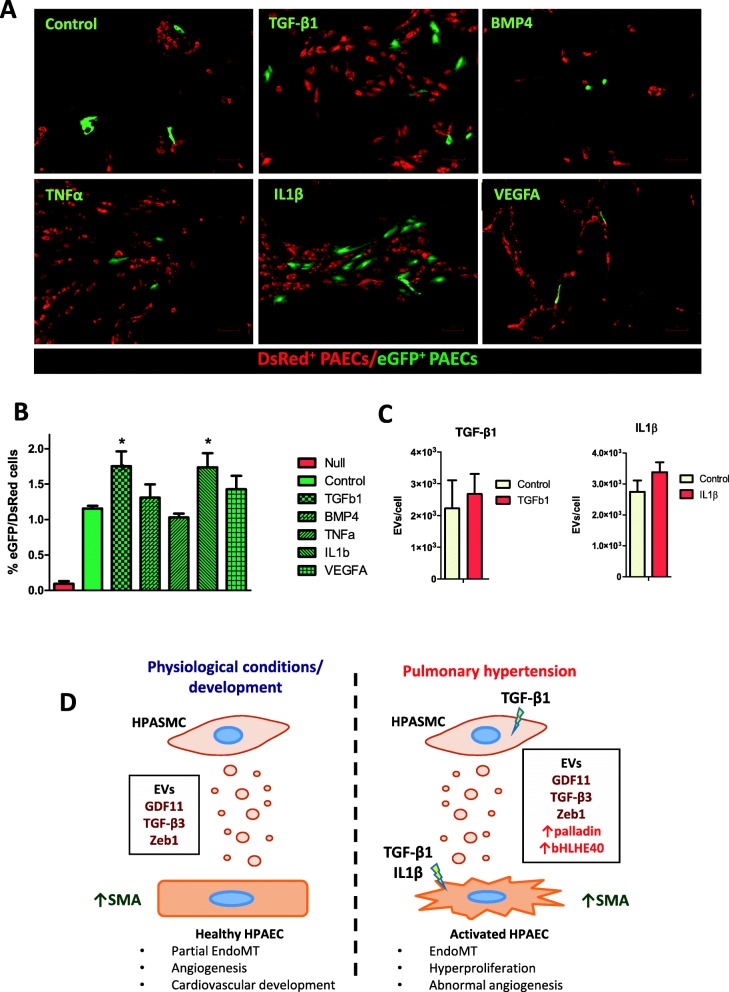
EV-mediated transfer of mRNA from HPASMC to HPAECs is enhanced by TGF-β1 and IL-1β. **a**, Our primary cell Cre-loxP system was used to analyse the alteration of uptake and translation of EV-mRNA by activated HPAECs in vitro. We analysed 5 cytokines related to PAH: TGF-β1 (5 ng/ml), BMP4 (5 ng/ml), IL-1β (5 ng/ml), TNF-α (1 ng/ml) and VEGFA (20 ng/ml). Stimuli were renewed every 48 h during the 7 days protocol. **b**, Quantification of Cre-induced recombination by FACS showed significant differences in TGF-β1 and IL-1β treated HPASMC Cre+: HPAECs Rep+ co-cultures as compared to the untreated. **c**, NTA showed no alteration of release of EVs/cell to supernatant in HPASMCs treated with TGF-β1 and IL-1β (both 10 ng/ml, 48 h and renewed every 24 h). **d**, Schematic representation of the biological relevance of the achieved results. Data are presented as mean ± SEM. *P ≤ 0.05, **P ≤ 0.01, ***P ≤ 0.001 (Student’s t-test). All procedures were carried out with n = 3 biological replicates. Scale bars 4x = 300 μm; 10x = 120 μm

Using this methodology, we next investigated whether EV-mediated communication from HPASMC-to-HPAEC was altered by excessive TGF-β signalling, as well as by other cytokines involved in PAH including IL-1β, TNF-α and VEGFA, which have been found increased in the serum of PAH patients [30, 31]. Results showed significant variation in transfer of EV-RNA with TGF-β1 and IL-1β, whereas BMP4, TNF-α and VEGFA showed no differences (Fig. 6a and b). NTA analysis of EVs released by HPASMCs upon stimulation with TGF-β1 and IL-1β showed no significant alteration of EVs/cell in the conditioned media (Fig. 6c). Hence, TGF-β1 and IL-1β enhance transport of EVs from HPASMCs to HPAEC but do not increase release by the former, which implies that the observed enhanced communication is mainly due to augmented uptake by activated HPAECs.

## Discussion

In this study, we investigated the RNA diversity in EVs from healthy HPASMCs and its variation in 2 different conditions of altered TGF-β signalling. Untreated EVs showed a differential transcriptome of that of HPASMCs. Among the enriched cargoes in untreated EVs we found GDF11 and TGF-β3, both ligands of the TGF-β superfamily, and Zeb1. Besides, we found an upregulation of palladin and bHLHE40 with TGF-β1. Using a novel primary cell suitable Cre-loxP method we demonstrated functional EV-mediated transport of mRNA from HPASMC-to-HPAEC in normal conditions and an enhanced uptake by activated HPAECs.

Our study shows the potential for these EVs to have a paracrine effect on TGF-β signalling and hence play a relevant role in vascular physiology. Furthermore, they could also contribute to PAH development since excessive TGF-β stimulation has been proven to underlie this pathology [2]. TGF-β3 has been previously found to be enriched in EVs from mesenchymal stem cells (MSCs) [32]. We then analysed the protein expression of both TGF-β ligands, finding that this enrichment was only evident for the mRNA as CD63^+^ EVs didn’t co-localize with both cargoes at the protein level.

The transcription factor Zeb1, a well-known gatekeeper for EMT and EndoMT [33], was also enriched in HPASMC-EVs. This was suggesting a potential contribution of HPASMC-EVs in EndoMT processes taking place in neighbouring HPAECs. Although the presence of Zeb1 mRNA has been also described in exosomes derived from mesenchymal non-small cell lung cancer cells [34], this is the first time the enrichment of this transcription factor in HPASMC-EVs has been demonstrated and puts these EVs in the spotlight as potential contributors for EndoMT, thus they might play an important role in vascular remodelling occurring during development. This is reinforced by co-localization with CD63 in HPASMCs and western blot analysis showing a specific expression of Zeb1 protein in EVs, which implies enrichment in EVs at both mRNA and protein levels. The localisation of mRNA and protein from the same gene in the vesicle is unexpected, since the mechanisms of sorting into EVs will be different for both types of macromolecules. In this respect, the enrichment of Zeb1 RNA and protein in EVs is quite remarkable. Even under basal conditions, HPASMCs show enrichment in many different RNA species, a number of which are known mediators of important biological effect affecting the endothelium.

Next, we studied the molecular effects of TGF-β modulation in both HPASMCs and their EVs. Many extracellular matrix genes were upregulated in HPASMCs upon stimulation supporting a switch into a pro-fibrotic phenotype. Interestingly, a considerable amount of inflammatory genes were found downregulated. This for the first time reveals that excessive TGF-β signalling promotes fibrosis without activating inflammatory pathways in HPASMCs. It is well-known that there is a highly inflammatory milieu in the pulmonary artery during PAH coming from infiltrated lymphocytes and other resident cell types [35]. It is therefore likely that other stimuli present in the vessel are responsible for the inflammatory response of HPASMC during PAH.

Then we compared the cargoes altered in HPASMCs-EVs in response to TGF-β stimulation. Upregulation of transcripts involved in actin and ECM remodelling by TGF-β1 was found in EVs according to the GO enrichment analysis. Thus, these EVs show a potential to activate and induce phenotypic changes on HPAECs during PAH development if uptake was to be proven. Among these differential RNAs from TGF-β1-EVs treated HPASMCs, palladin and bHLHE40 (also called Dec1), which have been shown to promote a malignant pro-proliferative phenotype in cancer cells by promoting epithelial-to-mesenchymal transition [36–39], were further validated. The gene bHLHE40 is besides a downstream target of TGF-β signalling [40].

Since our transcriptomic analyses have suggested that HPASMC-EVs could have the potential to affect HPAECs in physiological and pathological conditions, we aimed to study the potential ability of these EVs to transfer mRNA cargo to neighbouring HPAEC. To do so, we developed a novel Cre-loxP methodology suitable to primary cells with limited lifespan in vitro. This paves the way for future studies using our refined system in cell cultures requiring a shorter procedure as well as offering results in half the time in all cases. With our improved method, we have been able to visualise for the first time functional communication from HPASMC-to-HPAEC in vitro, demonstrating transfer and translation of Cre mRNA through EVs, resulting in protein expression in recipient HPAECs. Thus, mRNAs transported to HPAECs may lead to functional activation. To test this, we analysed two markers of EndoMT and vascular remodelling, smooth-muscle actin and collagen III, on HPAECs Rep^+^ after 7 days co-culture with HPASMCs Cre^+^. We observed a dramatic increase of SMA-positive HPAECs (13-fold) on cells that took up HPASMC-EVs after co-culture, compared to those that did not. This result evidences that HPASMC-EVs promote a phenotypic switch of HPAECs, possibly contributing to induce EndoMT. We also analysed collagen III, as a marker of fibrosis, which is also increased in EndoMT. In this case the levels observed in HPAECs after co-culture with HPASMCs were high, regardless of the former having taken up EVs. Probably due to this, only a moderate close-to-significance (pvalue = 0.086) increase of positive cells was observed. It is probable that the cell:cell contact from HPASMCs and HPAECs could have resulted in the high expression of collagen III observed in the endothelial cells. Nevertheless, the observed augment of SMA^+^ HPAECs on those taking up HPASMC-EVs clearly shows that these vesicles are able to promote phenotypic changes in HPAECs under basal conditions. Since EVs show enrichment in well-known mediators of EndoMT, the observed increase points to an active contribution of EV-mediated HPASMC-to-HPAEC communication in this transition, which is critical for cardiovascular development and homeostasis [33].

We then investigated if there is an enhancement in this communication under pathological conditions, aiming to modulate uptake by HPAECs using cytokines found to increase in PAH: TGF-β1, BMP4, TNF-α, IL-1β and VEGFA [5, 30, 31]. This validates our newly optimised methodology for analysing the alteration of EV-mediated transport in primary cell culture models. It is known that IL1β and TNF-α are inflammatory. While the former participates in the pathogenesis of some forms of PAH [41], the latter contributes to it by interacting with TGF-β signalling [42]. VEGFA is a central cause for PAH development also found increased in diseased lungs and alteration of the signalling pathway triggered by its receptor using SU5416 is a widely used PAH animal model [43]. Results showed increased up-take and translation of EV-mRNA by both TGF-β1 and IL-1β stimulated HPAECs, since release of EVs by HPASMC stimulated with these cytokines was found unaffected. The observed enhancement of EV-mediated HPASMCs:HPAECs communication with TGF-β1 and IL-1β, together with the results that showed increase in SMA, suggest that these EVs shall play a relevant role in the transition of HPAECs into mesenchymal cells during pulmonary hypertension development.

Since GDF11 has been reported to promote proliferation and abnormal angiogenesis on HPAECs [44] and TGF-β3 to contribute to vascular remodelling occurring during PAH [45], the enhanced EV uptake observed might lead to functional changes in HPAECs towards a PAH phenotype.

The hereby-described paracrine signalling, potentially taking place in the pulmonary artery, point to both TGF-β dependent and independent mechanisms, since we show that HPASMC-EVs are able to induce a phenotypic switch of HPAECS in basal conditions. The contribution of TGF-β independent paracrine signalling mechanisms in regulating fibrosis during PAH has been already reported and HPAEC-EVs were shown to mediate it [46]. It is therefore likely that these TGF-β independent mechanisms play an important role in EV-mediated communication within the pulmonary artery. This would be the case for IL1-β, according to our results, in the same way that has been recently described for aldosterone [46].

## Conclusions

In summary, we have proven that HPAECs can take up HPASMC-EVs and efficiently translate their mRNA cargo. These EVs are enriched in Zeb1 and TGF-β superfamily ligands and contribute to induce EndoMT on HPAECs. The effects of HPASMC-EVs in healthy HPAECs might be part of the physiological regulation of TGF-β signalling and contribute to partial EndoMT, which has been shown to promote angiogenesis [47] and is critical for the cardiovascular system during development [33, 48]. We have shown that activated HPAECs increase uptake of HPASMC-EVs and therefore this mechanism might as well contribute to PAH development (Fig. 6d).

The results of these studies prove the biological relevance of EV-mediated communication from HPASMC to HPAECs and provide evidence of its potential role in PAH development, paving the road for future in vivo studies to examine their function further in physiological and pathological conditions.

## Supplementary information

**Additional file 1: Figure S1.** HPASMC treatment and low-input RNA-Seq QC. **A**, **B**, Quality of RNA from HPASMCs and EVs was checked with the Agilent Bioanalyzer. The total amount of RNA obtained from EVs was 9 ng, thus we utilised a low-input RNA-Seq approach. RNAseq library was prepared with SMARTer Low-Input Strand-Specific Total RNA-Seq for Illumina and NGS performed by BGI Tech Solutions on a HiSeq 4000. **C**, Number of paired-end reads were between 5.5 × 107–6 × 107 for all samples. **Figure S2.** Selection of EV-markers for immunocytochemistry. **A**, In order to detect EVs from HPASMCs by means of immunocytochemistry (ICC) we assayed 3 different EV markers: CD9, CD81 and CD63. Staining for CD9 or CD81 was mainly present on plasma membrane while EV-like structures showing positive staining were found within the cytoplasm and membrane of HPASMCs in the case of CD63. **B**, Main subcellular location found in Human Protein Atlas for these proteins were: membrane for both CD9 and CD81 and vesicle for CD63, which is consistent with the results obtained by ICC on HPASMCs. **Fig. S3.** Analysis of protein levels of GDF11 and TGF-β3 and colocalisation with CD63. Immunocytochemistry of GDF11 (**A**) and TGF-β3 (**B**) proteins together with CD63 was performed. Co-localization with CD63 was negative, with GDF11 being very minimally expressed on the protein level. **C**, Isotype negative Control. Scale bars = 10 μm. (PDF 8509 kb) **Fig. S2.** Selection of EV-markers for immunocytochemistry. **A**, In order to detect EVs from HPASMCs by means of immunocytochemistry (ICC) we assayed 3 different EV markers: CD9, CD81 and CD63. Staining for CD9 or CD81 was mainly present on plasma membrane while EV-like structures showing positive staining were found within the cytoplasm and membrane of HPASMCs in the case of CD63. **B**, Main subcellular location found in Human Protein Atlas for these proteins were: membrane for both CD9 and CD81 and vesicle for CD63, which is consistent with the results obtained by ICC on HPASMCs. **Fig. S3.** Analysis of protein levels of GDF11 and TGF-β3 and colocalisation with CD63. Immunocytochemistry of GDF11 (**A**) and TGF-β3 (**B**) proteins together with CD63 was performed. Co-localization with CD63 was negative, with GDF11 being very minimally expressed on the protein level. **C**, Isotype negative Control. Scale bars = 10 μm.

**Additional file 2: Table S1.** Enrichment analysis of healthy HPASMC-EVs versus their donor HPASMCs. (XLSX 6647 kb)

**Additional file 3: Table S2.** RNA-Seq results and differential transcriptome analyses of HPASMCs treated with TGF-β1 and BMP4. (XLSX 7595 kb)

**Additional file 4: Table S3.** Gene Ontology enrichment analysis for Biological Process using differential RNAs found in TGF-β1 treated HPASMC-EVs. (XLSX 16 kb)

**Additional file 5: Table S4.** RNA-Seq results and differential transciptome analyses of HPASMC-EVs treated with TGF-β and BMP4. (XLSX 6597 kb)

**Additional file 6: Table S5.** Gene Ontology enrichment analysis for Biological Process using differential RNAs found in TGF-β1 treated HPASMC-EVs. (XLSX 17 kb)

**Additional file 7:** **Table S6.** List of primers used. (PDF 297 kb)

## Data Availability

The datasets generated and/or analysed during the current study have been deposited in NCBI's Gene Expression Omnibus and are accessible through GEO Series accession number GSE131998 (https://www.ncbi.nlm.nih.gov/geo/query/acc.cgi?acc=GSE131998).

## Abbreviations

bHLHE40: Class E basic helix-loop-helix protein 40
BMP4: Bone morphogenetic protein 4
EndoMT: Endothelial-to-mesenchymal transition
EVs: Extracellular vesicles
GDF11: Growth/differentiation factor 11
HPAEC: Human pulmonary arterial endothelial cells
HPASMCs: Human pulmonary arterial smooth muscle cells
Il1-β: Interleukin-1 beta
PAH: Pulmonary arterial hypertension
qRT-PCR: Gene expression quantitative Real Time-PCR
SEM: Standard error mean
TGF-β: Transforming growth factor beta
TNF-α: Tumor necrosis factor
VEGF: Vascular endothelial growth factor
Zeb1: Zinc finger E-box-binding homeobox 1
