# Supplementary material for: Extracellular vesicle cross-talk between pulmonary artery smooth muscle cells and endothelium during excessive TGF-β signalling: implications for PAH vascular remodelling
Source: Cell Commun Signal. 2019 Nov 8;17:143. doi: 10.1186/s12964-019-0449-9 (PMC6839246; doi:10.1186/s12964-019-0449-9)
Supplement: Supplementary file 1 — Additional file 1: Figure S1. HPASMC treatment and low-input RNA-Seq QC. A, B, Quality of RNA from HPASMCs and EVs was checked with the Agilent Bioanalyzer. The total amount of RNA obtained from EVs was 9 ng, thus we utilised a low-input RNA-Seq approach. RNAseq library was prepared with SMARTer Low-Input Strand-Specific Total RNA-Seq for Illumina and NGS performed by BGI Tech Solutions on a HiSeq 4000. C, Number of paired-end reads were between 5.5 × 10^7^–6 × 10^7^ for all samples. Figure S2. Selection of EV-markers for immunocytochemistry. A, In order to detect EVs from HPASMCs by means of immunocytochemistry (ICC) we assayed 3 different EV markers: CD9, CD81 and CD63. Staining for CD9 or CD81 was mainly present on plasma membrane while EV-like structures showing positive staining were found within the cytoplasm and membrane of HPASMCs in the case of CD63. B, Main subcellular location found in Human Protein Atlas for these proteins were: membrane for both CD9 and CD81 and vesicle for CD63, which is consistent with the results obtained by ICC on HPASMCs. Fig. S3. Analysis of protein levels of GDF11 and TGF-β3 and colocalisation with CD63. Immunocytochemistry of GDF11 (A) and TGF-β3 (B) proteins together with CD63 was performed. Co-localization with CD63 was negative, with GDF11 being very minimally expressed on the protein level. C, Isotype negative Control. Scale bars = 10 μm. (PDF 8509 kb) Fig. S2. Selection of EV-markers for immunocytochemistry. A, In order to detect EVs from HPASMCs by means of immunocytochemistry (ICC) we assayed 3 different EV markers: CD9, CD81 and CD63. Staining for CD9 or CD81 was mainly present on plasma membrane while EV-like structures showing positive staining were found within the cytoplasm and membrane of HPASMCs in the case of CD63. B, Main subcellular location found in Human Protein Atlas for these proteins were: membrane for both CD9 and CD81 and vesicle for CD63, which is consistent with the results obtained by ICC on HPASMCs. Fig. [file 12964_2019_449_MOESM1_ESM.pdf]

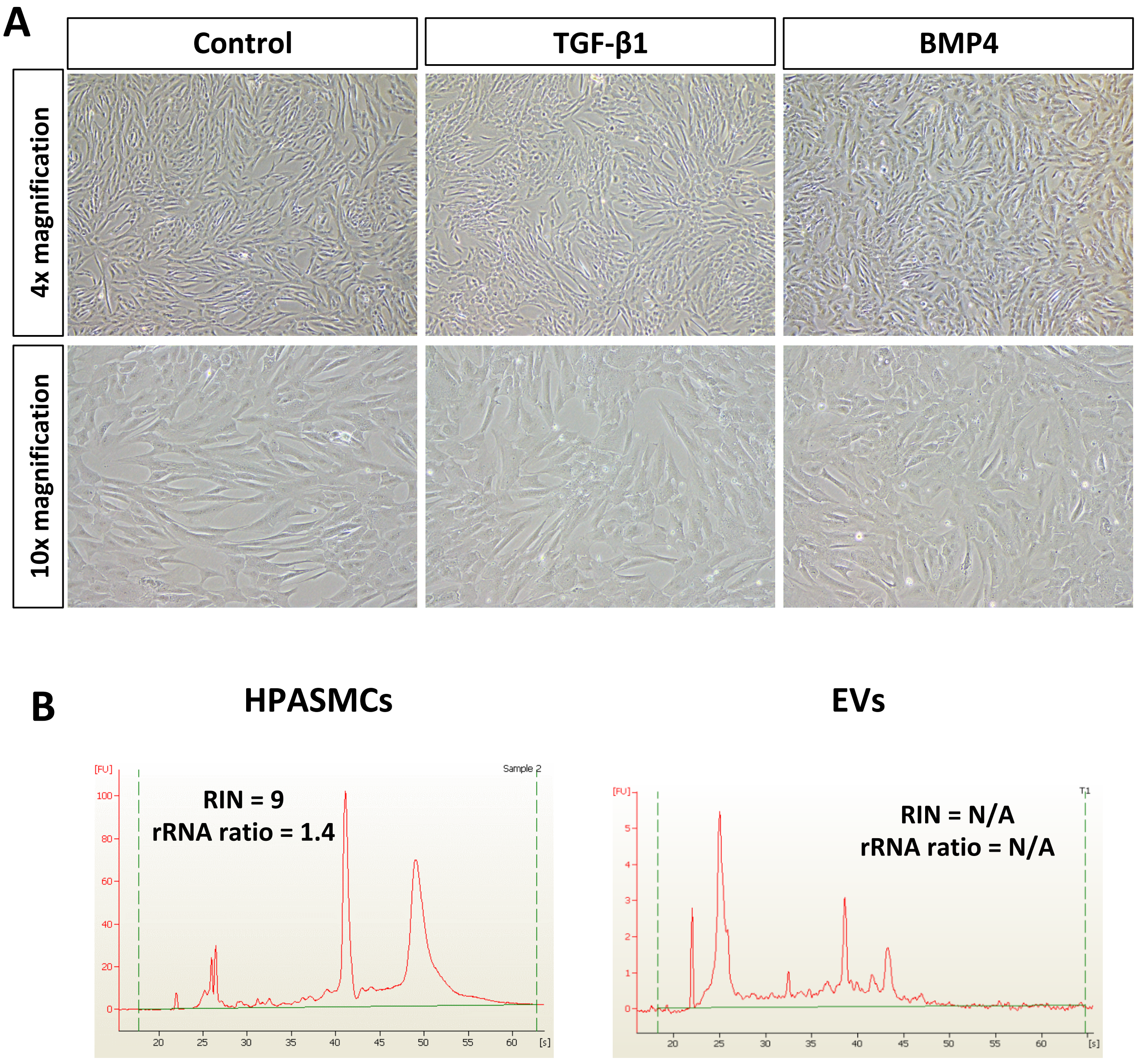

C

	number of paired-end reads	% read 1 left after removing duplicated reads	% read 2 left after removing duplicated reads
CT_EVs_2	55,345,844	3.25	10.66
CT_EVs_3	60,235,837	3.34	10.66
BMP_EVs_2	57,354,172	3.65	5.78
BMP_EVs_3	60,129,290	5.01	6.62
TGF_EVs_1	60,430,320	3.47	4.48
TGF_EVs_2	56,875,766	4.97	6.62
TGF_EVs_3	60,273,990	3.71	5.28
CT_Cells_1	60,071,538	15.53	14.35
CT_Cells_2	58,640,426	17.1	17.47
CT_Cells_3	59,267,958	16.76	17.09
BMP_Cells_1	60,338,541	15.53	17.98
BMP_Cells_2	60,041,129	15.91	18.09
BMP_Cells_3	60,559,555	18.48	19.9
TGF_Cells_1	60,470,881	15.36	17.64
TGF_Cells_2	60,550,755	20.23	38.1
TGF_Cells_3	58,602,528	16.5	30.83

Suppl Figure 1. HPASMC treatment and low-input RNA-Seq QC. A, **B**, Quality of RNA from HPASMCs and EVs was checked with the Agilent Bioanalyzer. The total amount of RNA obtained from EVs was 9 ng, thus we utilised a low-input RNA-Seq approach. RNAseq library was prepared with SMARTer Low-Input Strand-Specific Total RNA-Seq for Illumina and NGS performed by BGI Tech Solutions on a HiSeq 4000. **C**, Number of paired-end reads were between 5.5 x 107 - 6 x 107 for all samples

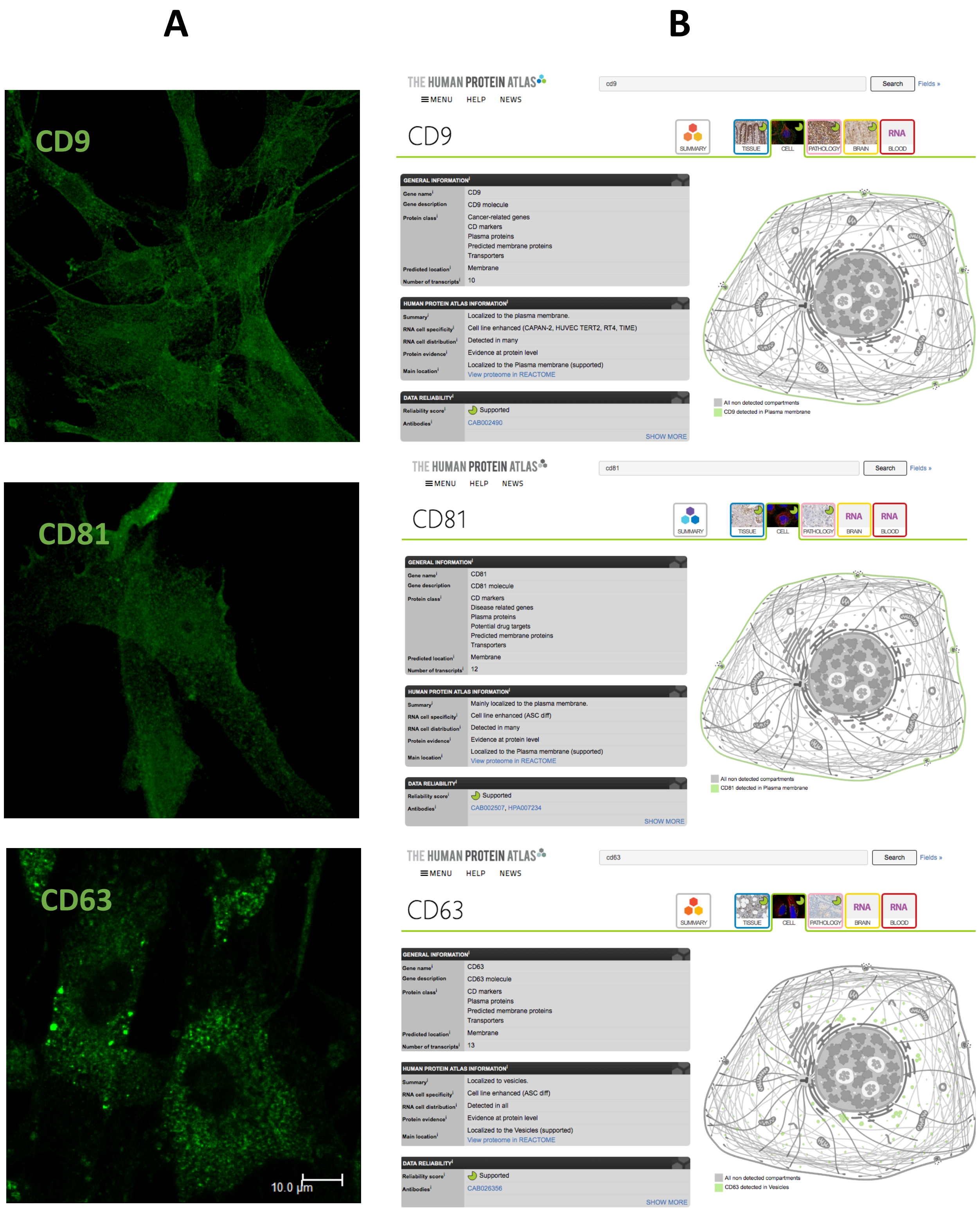

Suppl Figure 2. Selection of EV-markers for immunocytochemistry. A, In order to detect EVs from HPASMCs by means of immunocytochemistry (ICC) we assayed 3 different EV markers: CD9, CD81 and CD63. Staining for CD9 or CD81 was mainly present on plasma membrane while EV-like structures showing positive staining were found within the cytoplasm and membrane of HPASMCs in the case of CD63.

B, Main subcellular location found in Human Protein Atlas for these proteins were: membrane for both CD9 and CD81 and vesicle for CD63, which is consistent with the results obtained by ICC on HPASMCs.

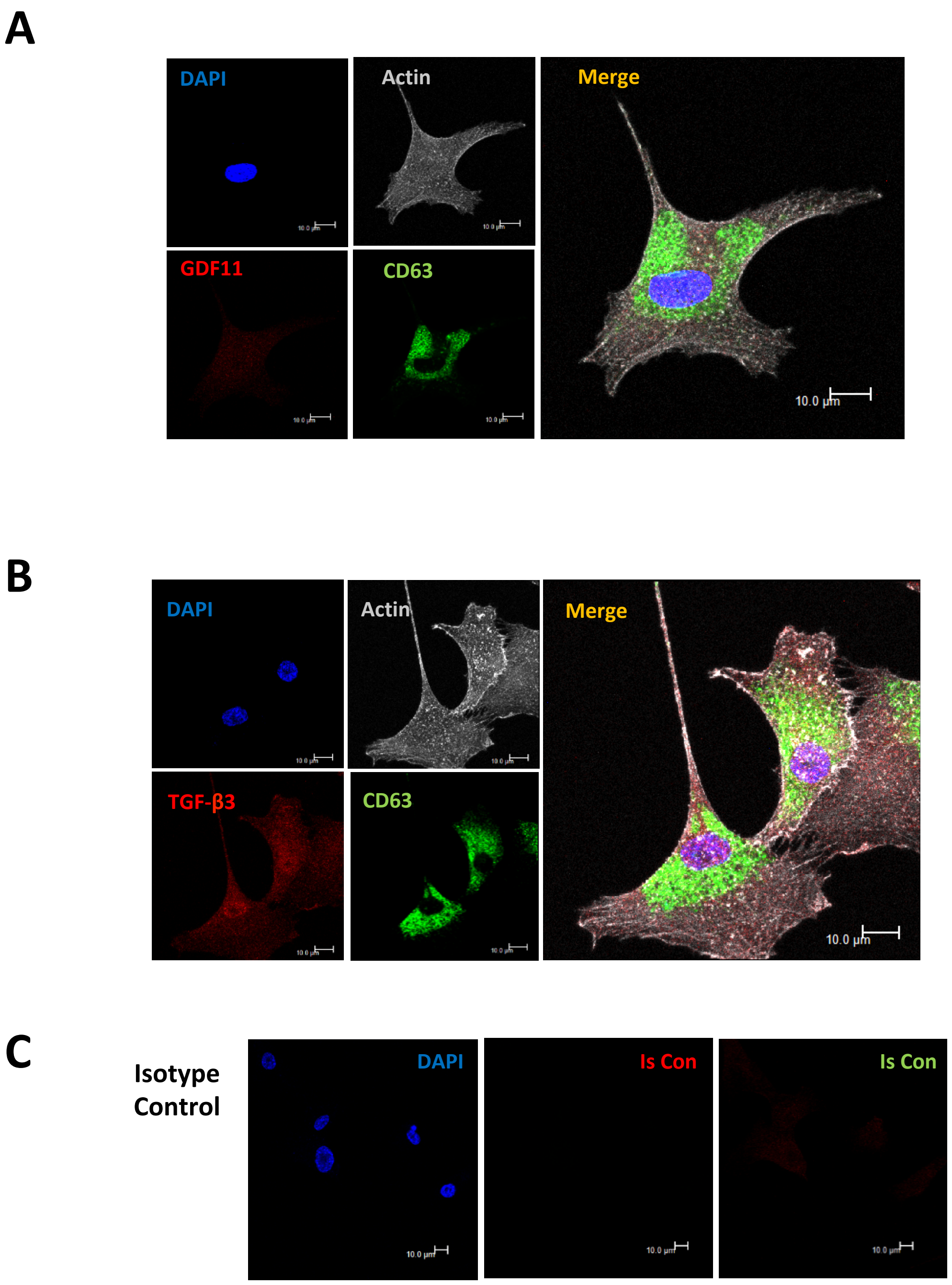

Suppl Figure 3. Analysis of protein levels of GDF11 and TGF-β3 and colocalisation with CD63. Immunocytochemistry of GDF11 (**A**) and TGF-β3 (**B**) proteins together with CD63 was performed. Co-localization with CD63 was negative, with GDF11 being very minimally expressed on the protein level. **C**, Isotype negative Control. Scale bars = 10 µm.
