# Supplementary material for: Extracellular vesicle cross-talk between pulmonary artery smooth muscle cells and endothelium during excessive TGF-β signalling: implications for PAH vascular remodelling
Source: Cell Commun Signal. 2019 Nov 8;17:143. doi: 10.1186/s12964-019-0449-9 (PMC6839246; doi:10.1186/s12964-019-0449-9)
Supplement: Supplementary file 7 — Additional file 7: Table S6. List of primers used. (PDF 297 kb) [file 12964_2019_449_MOESM7_ESM.pdf]

TaqMan Gene Experssion Assay

Target	Gene name	TaqMan Probe
**GDF11**	GDF11	Hs00195156_m1
**TGF-β3**	TGFB3	Hs01086000_m1
**Zeb1**	ZEB1	Hs01566408_m1
**bHLHE40**	BHLHE40	Hs01041212_m1
**Palladin**	PALLD	Hs00363101_m1
**Serpine1**	SERPINE1	Hs01126606_m1
**Id1**	ID1	Hs03676575_s1
**Id3**	ID3	Hs00171409_m1

TaqMan primer/probe

Target	Gene name	Forward primer	Reverse primer	Probe
		TGTGTGCCCGTCTGTTGTGT	GAGTCCTGCGTCGAGAGAGC	5'-(FAM)-CAGTGGCGCCCGAACAGGGA-(TAMRA)-3'

SYBR

Target	Gene name	Forward primer	Reverse primer
**Cre**	CRE	ATACCGGAGATCATGCAAGC	TTGCCCCTGTTTCACTATCC
